# Impact of Chlorinated-Assisted Backwash and Air Backwash on Ultrafiltration Fouling Management for Urban Wastewater Tertiary Treatment

**DOI:** 10.3390/membranes11100733

**Published:** 2021-09-27

**Authors:** Jiaqi Yang, Mathias Monnot, Lionel Ercolei, Philippe Moulin

**Affiliations:** 1 Laboratoire de Mécanique, Modélisation et Procédés Propres, Equipe Procédés Membranaire (EPM-M2P2-CNRS-UMR 7340), Aix-Marseille Univ., Europôle de l’Arbois, BP 80, Bat. Laennec, Hall C, CEDEX 04, 13545 Aix-en-Provence, France; jiaqi.yang@centrale-marseille.fr (J.Y.); mathias.monnot@univ-amu.fr (M.M.); 2 Société des Eaux de Marseille Métropole, 25 Rue Edouard Delanglade, BP 29, CEDEX 06, 13006 Marseille, France; lionel.ercolei@eauxdemarseille.fr

**Keywords:** NaClO-assisted backwash, UF membrane, irreversible fouling, permeability, membrane properties

## Abstract

To improve membrane fouling management, the NaClO-assisted backwash has been developed to improve permeability maintenance and reduce the need for intensive chemical cleanings. This study is aimed to focus on the efficiency of NaClO-assisted backwash in real UF pilot scale and with periodic classic backwash (CB) and air backwash (AB). The impacts on hydraulic filtration performance, physicochemical properties of membrane material under different addition frequencies of NaClO, and the performance of chlorinated CB and AB will be discussed. In result, 10 mg Cl_2_ L^−1^ NaClO addition in backwash water is confirmed to greatly improve the overall filtration performance and backwash cleaning efficiency. One condition stands out from the other due to better control of irreversible fouling, less NaClO consumption in 10 years prediction, sustainable and adaptable filtration performance, and less potential damage on the physicochemical properties of the membrane. Additionally, it can be inferred from this experiment that frequent contact with NaClO induced some degradation on the PES-made UF membrane surface properties. To retain the best state of UF membrane on anti-fouling and qualified production, the optimized condition with more frequent NaClO contact was not suggested for long-term filtration.

## 1. Introduction

Over the last decades, the application of membrane technology on wastewater treatment has expanded rapidly under increasing stringent legislation and environmental protection requirements [1]. Particularly, ultrafiltration (UF) as tertiary wastewater treatment technology has been accepted broadly for non-potable wastewater reuse [2]. During filtration, while the optimization of UF operating parameters can control membrane fouling, membrane cleaning is fundamental for fouling removal. Physical backwashes and chemical cleanings are the commonly used methods to remove membrane fouling. Physical backwashes, such as classic backwash (CB) or air backwash (AB), alternates the shear forces on the membrane surface by air scouring, air injection, or water backflush, in order to loosen and dislodge the deposits [3]. However, physical backwash is mostly powerless on irreversible fouling removal during long-term filtration, and the residual foulants after backwashes would be recompressed after multi-cycle filtration [4]. The conventional chemical cleaning plays an important role on irreversible fouling separation by chemical reagents soaking/reacting with membrane fouling which can damage the foulant–foulant and membrane–foulant interactions [5]. However, using high concentrated acids, soda, and/or oxidants/disinfectants and longer soaking time will gradually cause irreversible damages on membrane properties and filtration performances in long-term operation [3,6,7]. In this case, a novel chemically assisted maintenance backwash (CAMB), the combined physical backwash with lower concentrated oxidants/disinfectants (compared to chemical cleaning) in backwash water, has recently been developed for permeability maintenance improvement, so as to reduce the need for intensive chemical cleanings [8,9].

Sodium hypochlorite (NaClO), unproperly often called “chlorine”, is one of the commonly used oxidants/disinfectants, which can inactivate the microorganisms and oxidize the organic foulants to be more hydrophilic and easier to detach from the membrane surfaces [10,11,12]. Studies have also found that the presence of ClO^–^ is effective in terms of extracellular polymeric substances (EPS) disruption and microbial cell damage [13]. Researchers usually use the concentration of total chlorine or free chlorine to illustrate the quantity of chlorine they add in solutions [14]. In this study, the commonly mentioned chlorine concentration refers to total concentration unless otherwise specified as free chlorine. NaClO is an oxidant which is unstable under atmosphere with high temperature, light, strong acid, or other reductants. NaClO is also a strong base and weak acid salt that can hydrolyze and produce HOCl, NaOCl + H_2_O → Na^+^ + HOCl + OH^–^. The microbicidal activity of chlorine is largely attributed to undissociated HOCl. The dissociation of HOCI to the less microbicidal form (OCl^–^) depends on pH. The disinfecting efficacy of chlorine decreases with increasing pH, which is parallel to the conversion of undissociated HOCI to OCl^–^ [15,16]. Fukuzaki et al. [17] reported the bactericidal activity of 2.38 mg Cl_2_·L^−1^ (free chlorine) NaClO was increased with pH decreasing from 9.3 to 5.7 because the HOCl amount was increased from 13% (pH 9.3) to 98% (pH 5.7). In conventional chemical cleanings, NaClO is usually used at high levels (300–3000 mg NaClO·L^−1^/286–2860 mg Cl_2_·L^−1^) and in long time soaking for powerful membrane permeability recovery and irreversible fouling removal [8,18,19]. However, a high dosage of NaClO will accelerate the adverse effects on membrane properties and performance [19], by-products formation, and higher operating costs [9,20]. If it is in membrane bioreactor (MBR), the high level use of NaClO will inevitably suppress the microbial proliferation and the formation of sludge flocs [21,22]. Normally, the accumulated amount of NaClO in contact with membranes can be expressed as a total dose calculated in total chlorine concentration multiplied by contact time (CT) which can provide reference for the degradation progress of membranes. Hanafi et al. reported HClO and ·OH are the responsible species for the chain scission of polysulfone, polyamide, and polyethersulfone (PES) membranes [23,24,25,26]. Therefore, the accumulated amount of NaClO in membranes can cause degradation of membrane materials and membrane aging. PES membranes are considered highly tolerant to oxidants [27]. Wienk et al. [28] detected the aging of PES/PVP membrane under exposure to 6000 ppm/day of NaClO (approximately equal to 5718 mg Cl_2_·L^−1^·day). Yadav et al. [29] observed the PES membrane surface damage under exposure to 10,000–25,000 ppm/day of NaClO (approximately equal to 9530–23,853 mg Cl_2_·L^−1^·day), and the damage became worse with increasing amount of NaClO under the same conditions (temperature, pH, etc.). While ultrafiltration is mechanically and chemically stressed, the combined oxidant with physical backwash may accelerate physical damage and/or chemical degradation of membrane materials [30].

According to Fukuzaki et al. [31], low concentrations of OCl^−^ could destruct the exoplasmic organic matrix, while high concentrations of OCl^−^ led to diffusions of OCl^−^ into cells and disruption of cell metabolisms. Therefore, the new chemically assisted maintenance backwash first proposed by Wang et al. [8] with low dose of chlorinated disinfectants was developed and investigated on membrane cleaning efficiency. The effectiveness of the combined backwash with low dosage (<286 mg Cl_2_·L^−1^) of NaClO on membrane fouling removal has been mentioned in several publications (Table 1). Some studies added low level of NaClO into backwash water for disinfection but without special research on the impact of NaClO addition on cleaning efficiency, such as Wang et al. [32], and Liu et al. [33]. In 2001, Decarolis et al. [34] demonstrated that backwashing with chlorine addition (23.8 mg Cl_2_ L^−1^) significantly improved UF membrane productivity. Afterwards, Wang et al. [8] investigated the impacts of NaClO-assisted backwash on the hydraulic filtration performances of a membrane bioreactor (MBR) under different NaClO loads. Yue et al. [9] did a similar study scheme as Wang et al. [8] but with a different bioreactor, which was an anaerobic ceramic membrane bioreactor. In result, they both confirmed that backwash with a low level of NaClO enhanced the organic foulant degradation and inhibited microbial regrowth on membranes. Wang et al. [8] also mentioned that the NaClO-assisted backwash at lower NaClO concentrations and higher backflush frequencies made less adverse effects on the functional groups of the active layer of the membranes. Furthermore, Zhang et al. [35] stated that 100 mg·L^−1^ NaClO solution exhibited the best performance in removing the irreversible fouling resistance (88.4% ± 1.1%) from the algal-fouled UF membrane, compared to the solutions with 500 mg·L^−1^ NaOH, 500 mg·L^−1^ HCl and 150 mg·L^−1^ ethylenediaminetetraacetic acid. This might be attributed to the fact that NaClO could eliminate major foulants such as carbohydrate-like and protein-like materials on the membrane surface. In result, the cases in Table 1 confirmed the cleaning effectiveness of disinfectants (mostly NaClO) addition in backwash, but their concentrations varied under different conditions. 

The membrane foulants in municipal secondary effluent include organic fouling, inorganic fouling, and microbial fouling [3,36,37]. Organic fouling is the main cause of irreversible fouling for membranes. Nonetheless, the efficiency and practicality of NaClO-assisted backwash has not been studied in real UF pilot scale and even less with periodic classic backwash (CB) and air backwash (AB). Therefore, this study aims to investigate the influence of NaClO-assisted CB and/or AB on the hydraulic filtration performance and fouling management in a UF pilot plant with maximum flow rate of 20 m^3^·d^−1^. Additionally, the impacts on physicochemical properties of membrane material under different addition frequencies of NaClO and the performance of chlorinated CB and AB will also be discussed

## 2. Materials and Methods

### 2.1. Semi-Industrial UF Pilot Plant 

**Figure 1:**
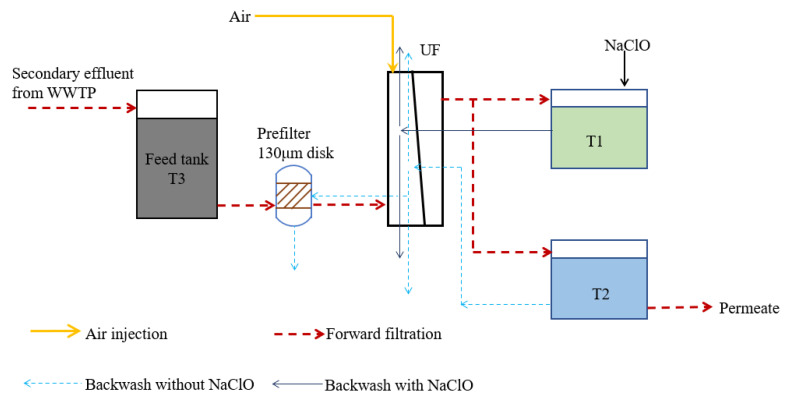
Diagram of semi-industrial pilot plant.

The semi-industrial UF pilot plant is automatically controlled and has a nominal capacity of 20 m^3^·d^−1^ [39]. Figure 1 shows the flow diagram of the pilot plant with filtration route and backwash routes. The feed water is the secondary effluent of a municipal wastewater treatment plant (WWTP) located in Châteauneuf-les-Martigues, France. The WWTP uses a conventional activated sludge process to treat raw wastewater. T1 and T2 are the tanks for permeate. Tank T1 contains permeate with chlorine in constant concentration. The chlorine concentration is controlled by the cooperation of control system and the chlorine meter. T2 is the permeate tank connected to the outside without chlorine used both as water production and backwash water. A 130 μm disk prefilter is used to avoid clogging of the hollow fibers. To control the filtration conditions and detect the filtration performance, pressure, temperature, chlorine and turbidity sensors of feed, pH sensor of effluent, and flowmeters are all connected to the data logger. The chlorine probe is a Dulcotest Type CGE 2-mA-10 ppm (ppm: parts per million, equivalent to mg·L^−1^ in water) from ProMinent (Dosiertechnik GmbH, Heidelberg, Germany) measuring free chlorine and organically bound chlorine (to cyanuric acid in the form of trichloroisocyanuric acid or sodium dichloroisocyanurate-bound chlorine). Each parameter is recorded every minute.

The membrane module is an ALTEON^TM^ I (Aquasource, Toulouse, France) multichannel hollow fiber UF module. The detailed information of the UF module is shown in Table 2. PES membrane is blended with polyvinylpyrrolidone (PVP) additives to increase the hydrophilicity for anti-fouling [23]. PES membranes are considered highly tolerant to oxidants (>250,000 mg Cl_2_ L^−1^ h) and to a wide pH range between 2 and 12 [27]. It is operated in dead-end filtration mode with an inside-out configuration. The operational transmembrane pressure (TMP) on UF is better to be controlled under 1 bar. The hydraulic resistance of new membrane module was measured with pure water to be 4 × 10^11^ m^−1^ at 20 °C (pure water permeability = 900 L·m^−2^·h^−1^·bar^−1^). The maximum permeability of UF membrane when filtrated with the feed water after deep chemical cleaning is around 650 L·m^−2^·h^−1^·bar^−1^ at 20 °C. Therefore, each filtration condition was started from initial Lp at around 650 L·m^−2^·h^−1^·bar^−1^, and the corresponding membrane resistance of 5.51 × 10^11^ m^−1^ at 20 °C was taken into account for future calculations.

The feed water quality is shown in Table 3, and the UF feed water contains bacteria, virus, organic matter and suspended solids.

### 2.2. Membrane Cleaning

Two modes of physical backwashes during filtration are classical backwashes (CB) and air backwashes (AB). A cleaning of CB includes 4 steps: top backwash, bottom backwash, both heads backwash, and prefilter backwash. AB includes air injection procedure followed with a CB cleaning. The air velocity during injection is around 0.6 m·s^−1^. The flow rate of physical backwash is around 2.5 m^3^·h^−1^, the backwash water velocity is 0.64 m·s^−1^. The detailed procedures of CB and AB are completely shown in Table 4. The different types of physical backwash are edited and represented with different serial numbers in the system to be easily distinguished. The physical backwashes without NaClO all use permeate from T2 (no chlorine tank) as backwash water. The physical backwashes with NaClO use permeate from T1 (tank with chlorine) as backwash water to clean internal membrane, and then use permeate from T2 as backwash water to backwash prefilter. The prefilter backwash as the final backwash step will always be backwashed with permeate from T2 to drain the chlorine in the system pipes and UF module. The flow chart of physical backwashes can be found in Figure 1. The AB has shown their excellent efficiency to remove the fouling whatever the type of water [39,40,41]. Through comparison, one backwash sequence (CB CB CB AB) in all conditions has the same consumption of permeate volume, same energy demand for backwash and air injection, and duration for backwash, the differences among them was only the addition of NaClO or not. This condition (CB CB CB AB) was optimized in a previous study [39].

Before new operating conditions start or if the TMP achieve to 0.3 bar for 60 s, corresponding to permeability of 200 L·m^−2^·h^−1^·bar^−1^ at 20 °C, a chemical enhanced backwash (CEB) was conducted. A set of CEB include an acid CEB and an alkaline CEB. The CEB starts with chemical injection into membrane modules and then a soaking with chemicals to degrade foulants. Chemicals are either sulfuric acid ([H^+^] = 1000 mg·L^−1^), or sodium hydroxide ([OH] = 800 mg·L^−1^) and chlorine ([active Cl] = 50 mg Cl_2_·L^−1^). The duration of soaking is 1200 s for each CEB. In addition, a forward flushing happens in the middle of each filtration cycle for about 10 s where 2.0~3.0 m^3^·h^−1^ feed water flows fast from bottom to top of the UF module. The forward flushing can assist to decrease aggregation or dense attachment of particulates from the membrane surface [42].

### 2.3. Filtration Conditions

The experiments were in dead-end, automatically filtration under constant flux mode. Based on the previous study of Yang et al. [39], the optimized filtration condition of this UF pilot plant and adopted in this present paper is a flux at 60 L·m^−2^·h^−1^, a filtration cycle time at 60 min, and a backwash sequence of 3 classic backwashes followed by 1 air backwash. The variable in this study is the chlorine addition in backwash water or not. Four backwash conditions were investigated in this test, shown in Table 5. Each condition was conducted continuously for 5–7 days to obtain stable filtration performance. After each condition, several CEBs were operated manually to improve permeability (Lp) to reach around 650 L·m^−2^·h^−1^·bar^−1^, so as to maintain the similarly initial filtration state for the next filtration conditions. To be noted, the parameters including flux and Lp, that could be affected by the temperature, have been normalized to a standard temperature (20 °C) to account for viscosity fluctuations with these parameters. During experiment, the chronological order of conditions is NNNN, NNNY, YYYN and finally YYYY where Y and N indicates, respectively, the presence (Yes) or absence (No) of chlorine during a backwash.

The operation of the four conditions intermittently lasted for 6 months, from October 2020 to June 2021. Low NaClO consumption conditions NNNN and NNNY were tested from October 2020 to December 2020, higher NaClO consumption conditions YYYN and YYYY were mainly tested from March 2021 to June 2021. The operation of each condition was repeated for more than one month for repeated tests to obtain stable and reliable results, and filtration results were compared.

### 2.4. Analysis

#### 2.4.1. Irreversible and Reversible Fouling Resistance

According to Darcy’s law [43], hydraulic resistance of the fouled membrane was measured with Equation (1):

(1) $$R_{t}=R_{\mathrm{irr}}+R_{\mathrm{re}}+R_{m}=\frac{\mathrm{TMP}}{\mu \cdot J}=\frac{1}{\mu \cdot \mathrm{Lp}}$$

where, TMP is the transmembrane pressure (Pa), μ is the viscosity of permeate (Pa·s) and J is the applied flux (m·s^−1^). The total membrane resistance (R_t_) includes three parts of resistances (m^−1^): irreversible fouling resistance (R_irr_) which cannot be removed by backwashes, reversible fouling resistance (R_re_) which can be removed by backwashes and membrane resistance (R_m_).

The flux at 20 ℃ is calculated through Equation (2) [44]:

(2) $$J\left(20\;℃\right)=\frac{\mu \left(T\right)}{\mu \left(20\right)}\cdot J\left(T\right)=J\left(T\right)\;\cdot e^{\left[0.0239\cdot \left(20-T\right)\right]}$$

In this context, the resistance composition before backwash and after backwash was emphasized for comparison. In general, the Rirr is considered as the fouling resistance that cannot be removed by CB. Therefore, the resistance composition before the n^th^ backwash (at the end of the n^th^ filtration cycle) includes R_irr_ and R_re_, which can be shown as:

(3) $$R_{t-\mathrm{end}}\left(n\right)=R_{m}+R_{\mathrm{irr}-\mathrm{end}}\left(n\right)+R_{\mathrm{re}-\mathrm{end}}\left(n\right)$$

From definition, only R_irr_ is left on the membrane after backwash (at the beginning of next filtration cycle) without R_re_. If it is turn for n^th^ CB, the initial R_irr_ in the (n + 1) filtration cycle is the same as R_irr_(n), as

(4) $$R_{\mathrm{irr}-\mathrm{ini}}\left(n+1\right)=R_{\mathrm{irr}-\mathrm{end}}\left(n\right)=R_{t-\mathrm{ini}}\left(n+1\right)-R_{m}$$

Normally, during filtration with periodic CB, the loosened and residual foulants after CB would be recompressed according to Ye et al. [45], which results in regular increase of R_irr-ini_. However, the occurrence of AB will break the rules of R_irr-ini_ increase after CB. Generally speaking, the residual R_irr-ini_ after AB will be decreased because AB can provide higher cleaning efficiency on membrane fouling than CB on the same water treatment, as R_irr-ini-AB_ (n + 1) < R_irr-ini-CB_ (n). Therefore, to better understand the fouling resistance composition and variation before and after n^th^ backwash at AB (at the end of the n^th^ filtration cycle, n is an integer multiple of 4), the R_irr-end_(n) before AB will be estimated by making arithmetic series with the R_irr-end_(n-1), R_irr-end_(n-2), and R_irr-end_(n-3) before CBs (Equation (4)), the equations are as below. 

Before AB:

(5) $$R_{t-\mathrm{end}}\left(n\right)=R_{m}+R_{\mathrm{irr}-\mathrm{end}}\left(n\right)+R_{\mathrm{re}-\mathrm{end}}\left(n\right)$$

(6) $$R_{\mathrm{irr}-\mathrm{end}}\left(n\right)=R_{\mathrm{irr}-\mathrm{end}}\left(n-1\right)+0.5\cdot \left[\left(R_{\mathrm{irr}-\mathrm{end}}\left(n-1\right)-R_{\mathrm{irr}-\mathrm{end}}\left(n-2\right)\right)+\left(R_{\mathrm{irr}-\mathrm{end}}\left(n-2\right)-R_{\mathrm{irr}-\mathrm{end}}\left(n-3\right)\right)\right]$$

After AB:

(7) $$R_{\mathrm{irr}-\mathrm{ini}}\left(n\right)=R_{t-\mathrm{ini}}\left(n+1\right)-R_{m}$$

Otherwise, the variation of irreversible fouling versus integral turbidity during filtration can be an indicator to investigate the influence of backwash conditions on filtration performance as the function of the feed water quality. The relationship of R_irr-end_ variation at each filtration cycle (n) with feed water turbidity integration (Equation (8)) will be considered to investigate the influence of backwash sequence on fouling formation whatever the quality of feed water (represented by the turbidity).

(8) $$\;\frac{R_{\mathrm{irr}-\mathrm{end}}\;\left(n\right)}{R_{\mathrm{irr}-\mathrm{end}}\left(1\right)}=f\left(\sum \int \mathrm{Tur}\;\mathrm{dt}\right)\;$$

Because the relationship here is mainly to investigate the backwash efficiency, therefore the filtration period should be without CEB. When a CEB occurs, the calculation of turbidity integrity should be restarted from 0. Already used to take into account the feed water quality [39], the turbidity integration with time is shown as follows with n = number of filtration cycle, and t_c_ the duration of a filtration cycle:

(9) $$\overset{n}{\underset{i=1}{\sum }}{\left[\int_{0}^{t_{c}}\mathrm{Turbidity}.\mathrm{dt}\right]}_{i}$$

#### 2.4.2. Reversibility

Filtration performance can be evaluated by parameters such as backwash effectiveness, fouling rate, and fouling resistance [46]. Backwash effectiveness can be indicated by fouling reversibility which was calculated after each filtration cycle (n) according to [47,48]. Reversibility after each filtration cycle could then be calculated using the initial TMP and final TMP values (${\mathrm{TMP}}_{\mathrm{end}}^{n}{\;\mathrm{and}\;\mathrm{TMP}}_{\mathrm{ini}}^{n}$) of the cycle (n) as well as the initial TMP of the next filtration cycle (${\mathrm{TMP}}_{\mathrm{ini}}^{\left(n+1\right)}$). As described above, the flux and viscosity at 20 °C are both constant during each filtration condition. Therefore, the reversibility can be calculated as follows in Equation (10):

(10) $$\mathrm{Reversibility}\left(n\right)=\frac{{\mathrm{TMP}}_{\mathrm{end}}^{n}-{\mathrm{TMP}}_{\mathrm{ini}}^{\left(n+1\right)}}{{\mathrm{TMP}}_{\mathrm{end}}^{n}-{\mathrm{TMP}}_{\mathrm{ini}}^{n}}=\frac{R_{\mathrm{end}}^{n}-R_{\mathrm{ini}}^{\left(n+1\right)}}{R_{\mathrm{end}}^{n}-R_{\mathrm{ini}}^{n}}\;$$

#### 2.4.3. Recovery Rates through Mass Balances

In addition to fouling resistances and reversibility, the recovery efficiency of physical backwashes on retained turbidity and retained TOC are illustrated to further verify the effectiveness of physical backwashes. For turbidity, when a sample needs dilution, turbidity is calculated using the following relationship [49,50]:

(11) $${\mathrm{Tur}}_{0}\cdot V_{T_{0}}={\mathrm{Tur}}_{d}\cdot \left(V_{T_{0}}+V_{T_{d}}\right)$$

Tur_0_ is the turbidity of the undiluted sample in NTU; Tur_d_ is the turbidity of the diluted sample in NTU; V_Td_ is the content of purified water (0 NTU) in the dilution in mL; and V_T0_ is the content of sample water in mL. The relationship between turbidity and volume is considered to be linear.

For organic matter, the accumulation of dissolved organic matter was already integrated with filtration time in Lin et al. [51]. Therefore, the turbidity and TOC are considered as accumulated amounts in membrane during the filtration cycles, and the removal rates of these parameters can be calculated with the following equations based on the amount of substance balance.

(1) Turbidity recovery rate:
  (12) $$R_{\mathrm{Tur}}=\frac{\int \left({\mathrm{Tur}}_{\mathrm{Feed}}–{\;\mathrm{Tur}}_{\mathrm{permeate}\;}\right){\;\mathrm{dV}}_{0}}{\int \left({\mathrm{Tur}}_{\mathrm{BW}}–{\;\mathrm{Tur}}_{\mathrm{permeate}\;}\right){\;\mathrm{dV}}_{\mathrm{Bw}}}\;\cdot 100\%=\frac{\left({\mathrm{Tur}}_{\mathrm{Feed}}–{\;\mathrm{Tur}}_{\mathrm{permeate}\;}\right)\cdot J\cdot S\cdot t\;}{({\mathrm{Tur}}_{\mathrm{BW}}–{\;\mathrm{Tur}}_{\mathrm{permeate}\;})\cdot ∆V_{\mathrm{BW}}}\;\cdot 100\%$$
(2) TOC recovery rate:
  (13) $$R_{\mathrm{TOC}}=\frac{\int \left({\mathrm{TOC}}_{\mathrm{Feed}}–{\;\mathrm{TOC}}_{\mathrm{permeate}\;}\right){\;\mathrm{dV}}_{0}}{\int \left({\mathrm{TOC}}_{\mathrm{BW}}–{\;\mathrm{TOC}}_{\mathrm{permeate}\;}\right){\;\mathrm{dV}}_{\mathrm{Bw}}}\;\cdot 100\%=\frac{\left({\mathrm{TOC}}_{\mathrm{Feed}}–{\;\mathrm{TOC}}_{\mathrm{permeate}\;}\right)\cdot J\cdot S\cdot t\;}{({\mathrm{TOC}}_{\mathrm{BW}}–{\;\mathrm{TOC}}_{\mathrm{permeate}\;})\cdot ∆V_{\mathrm{BW}}}\;\cdot 100\%$$

Here, V_0_ is the filtration volume through the UF membrane during one filtration cycle, L. V_BW_ is the physical backwash volume during one physical backwash, V_BW_ = 36 L. J is the filtration flux, J = 60 L·h^−1^·m^−2^, S is the surface area of UF membrane, S = 9 m^2^ and t is the filtration time during one filtration cycle, t = 1 h.

#### 2.4.4. Statistical Test

Statistical analysis was used to determine if there was a statistically significant linear regression between backwash reversibility and Rirr before backwash. The hypotheses were analyzed by XLSTAT (Addinsoft, New York, NY, USA), ANOVA test.

### 2.5. Water Quality Assessment

The turbidity of UF feed was measured and recorded every minute of the pilot plant using a probe TurbiMax W CUS31(Endress Hauser, Nesselwang, Germany). On sampling, the turbidity of UF feed, permeate, and backwashes was tested in laboratory with a turbidity meter (Turb 550 IR, WTW, Germany). A TOC-L machine (Shimadzu, Japan) based on the 680 °C combustion catalytic oxidation method was used to measure the concentration of total organic carbon (TOC). The non-purgeable organic carbon (NPOC) method with a detection limit of 4 μg·L^−1^ was used.

## 3. Results

### 3.1. Permeability Variation

**Figure 2:**
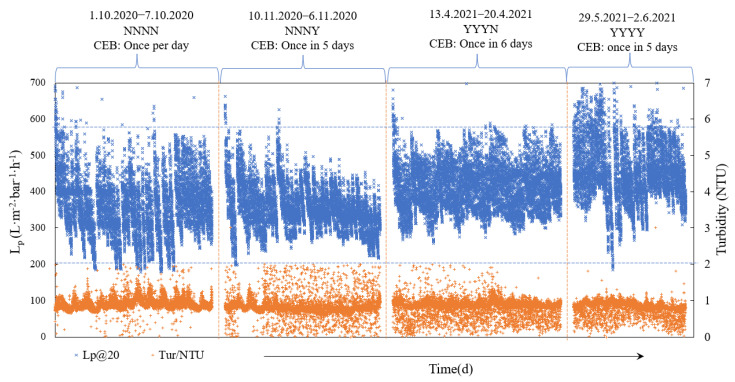
Permeability, feed water turbidity and temperature variation versus time in different backwash conditions.

During filtration, the permeability variation gives the hydraulic filtration performance of the UF membrane, as shown in Figure 2. According to Figure 2, the turbidity of UF feed was stabilized around 1 NTU in all conditions. However, the turbidity shows slight periodical variation with time in a day because the feed water quality seems positively correlated to the variations of raw wastewater flow rate as demonstrated in Yang et al. [39]. In addition, the temperature of the four conditions is 21 ± 2 °C and there is also a cyclical variation of temperature during the day and night. Nevertheless, the variation of feed water quality was undergone of all conditions, thus the feed water quality can be considered to have a similar effect on the four conditions. From CEB occurrences, condition NNNN resulted in the highest frequency of CEB, with 1 CEB per day. Additionally, condition YYYY had 1 CEB in 5 days, condition NNNY had 1 CEB in 6 days, and condition YYYN had 1 CEB in 6 days operation, but these three last conditions are similar. From permeability stability, condition NNNN showed the fastest decrease of permeability to 200 L·m^−2^·h^−1^·bar^−1^ which triggered CEB cleaning. Condition NNNY showed stable variation of permeability, the addition of NaClO only in AB largely improved the UF filtration performance compared to NNNN, with only 1 CEB occurrence in 6 days, and the CEB occurred at the 1st day of operation and no CEB occurred in the last 6 days. The reason for faster permeability decreases on the 1st day may be due to the variation of feed water quality, or that the newly cleaned membrane was sensitive to cake deposition which had a higher chance of pore blocking and lower reversibility of backwash. Similarly, Ye et al. [52] found less reversible fouling in the first few cycles of filtration of seawater than in the following cycles. In whole filtration, the permeability in condition NNNY showed a gradual downward trend. The permeability after stabilization in condition YYYN showed the most sustainable variation during one-week filtration, stabilized in 260–580 L·m^−2^·h^−1^·bar^−1^, while the permeability in condition NNNN, NNNY, and YYYY were stabilized on average around 200–500 L·m^−2^·h^−1^·bar^−1^, 230–580 L·m^−2^·h^−1^·bar^−1^, and 300–650 L·m^−2^·h^−1^·bar^−1^, respectively (some individual highs and lows and initial permeability are not taken into account). It seemed that the efficiency of physical backwashes in YYYN was just balanced to the accumulation of fouling rates, especially after 2 days operation, and the permeability after backwash could achieve to 100% recovery. So far, the addition of NaClO in either AB or CB could improve the membrane filtration performances greatly, including more sustainable permeability variation and less CEB occurrences.

The recovered permeability in condition YYYY after backwashes was higher than the others which could be achieved to 700 L·m^−2^·h^−1^·bar^−1^ in the first two days. The CEB in condition YYYY was triggered on the 2nd day of filtration, the fast decrease of permeability mainly occurred in 5–6 filtration cycles before CEB. Except the rapid permeability decrease on the 2nd day, the peak of permeability and average permeability were both higher than the other conditions. Considering that the variation of turbidity in that period was in regular ranges, the exceptional rapid decrease of permeability may be caused by other parameters that were not taken in account, such as the organic matter in the feed water, or low temperature.

A difference of the YYYY condition with the other three conditions was the peak permeability values. The UF membrane was strongly cleaned by CEB before each condition started which indicated that both the reversible and irreversible fouling can be removed greatly. The fouling formation in the first few cycles of each condition may be mainly because of pore blocking which is irreversible, thus the permeability decreased faster at the beginning of filtration than in the following cycles [45,53]; the physical backwash normally cannot recover the permeability back to the initial levels because of their poor separation strength on irreversible foulant. From Figure 2, it can be seen obviously the fast decrease of permeability at the first few cycles in conditions NNNN, NNNY, and YYYN, and even after automatic CEB, the permeability hardly reached 650 L·m^−2^·h^−1^·bar^−1^. In condition YYYY, the permeability could be easily recovered to the 700 L·m^−2^·h^−1^·bar^−1^ with all NaClO-assisted physical backwashes. Before condition YYYY, the pilot plant was just finished with the condition YYYN which already maintained the membrane in very good permeability variation. With NaClO addition both in CB and AB, it seems that the condition YYYY can reach to more than 100% recovery of the decreased permeability in a filtration cycle. The increase of permeability was also observed by Alhweij et al. and Wienk et al. [28,54]. The UF membrane showed quite good removal efficiency on parameters such as turbidity, microplastics, bacteria, virus, and organic matter of UF feed in all conditions, and the permeate quality was detected to be good enough to be reused in non-potable applications as it met reuse guidelines of the WHO [55], reuse standards of France [56], and the most recent EU regulation for agricultural irrigation [57].

To summarize the disinfection efficiency of NaClO, values for concentration multiplied by contact time (CT) considering backwash and CEB occurrences based on the above performance of each condition, measured in mg Cl_2_·L^−1^ ·h, were estimated for a simulated 10 years of operation (Table 6). The frequency of CEB occurrence here is estimated after feedback of this study as once per day for NNNN condition and similar for the others, once in 5 days, once in 6 days, and once in 5 days, respectively, for NNNY, YYYN, and YYYY conditions.

Referring to impacts of chlorinated disinfectants on membranes, many studies have reported that NaClO can attack the chemical bonds of polymeric membranes and degrade the membrane material due to long-term exposure or excessive dosage of chemicals [13,23,54,55,56,57,58,59]. Similarly, excessive chlorine can impact the PES membranes, but the degree of impact varies a lot in different studies. Some studies pointed out the occurrence of a PES-chain scission and PES material degradation though excessive chlorination, such as after 28,500 mg·L^−1^ total free chlorine of NaClO (27,160 mg Cl_2_·L^−1^) contact for 8 months [60], or after 200–2400 mg·L^−1^ total free chlorine of NaClO (190–2287 mg Cl_2_·L^−1^) contact for 10–60 d [23]. Fu and Zhang carried out the proposed PES degradation mechanisms that the PES may undergo a chain scission of the backbone structure into sulfonic acid groups and phenyl chloride groups [30]. Hanafi et al. [23] showed that exposure of PES/PVP membranes to NaClO even led to the appearance of macrovoids in the membrane sub-layer. On the contrary, some researchers found slight modification on PES membranes. Alhweij et al. [54] found that only slight changes in PES surface roughness was observed after exposure to 238 mg Cl_2_·L^−1^ of NaClO for 3 days. The permeability of PES membrane increased by 74% after chlorine exposure, but SEM images confirmed that there were no observed structural damages on the membrane. Wypysek et al. [61] also stated that a damaged separation skin does not have a significant negative influence on filtration performance of PES membrane. Yadav et al. [29] also mentioned that the changes of PES membrane surface properties was observed after exposure to 10,000–25,000 mg·L^−1^·day of NaClO (9530–23,825 mg Cl_2_·L^−1^·day) at pH 9–12, but no great changes were observed on membrane mechanical properties and tensile strength. However, according to Wang et al. [62], long time exposure to chlorine could impact physical and chemical properties of membranes regardless of the chlorine concentration. This phenomenon can be explained by the study of Abdullah and Bérubé [63], who stated that the more accurate relationship of chemical exposure to membranes should be C^n^T, not the CT; “n” is the power coefficient and was determined <1 from their experiment, which indicated that the contact time of cleaning had a more severe impact on the changes in the physical/chemical characteristics of the membranes than the NaClO concentration. From Table 6, condition NNNY resulted in the lowest CT of NaClO and equivalent consumption per m^3^ of permeate compared to the others, while condition NNNN resulted in the highest. Although the CT of NaClO in condition YYYY was lower than that of NNNN, YYYY provided higher contact frequency of NaClO to membranes. Therefore, it is not recommended to apply either YYYY or NNNN condition for long-term filtration purposes in case of the irreversible changes on the membrane.

### 3.2. Fouling Formation Mechanism

**Figure 3:**
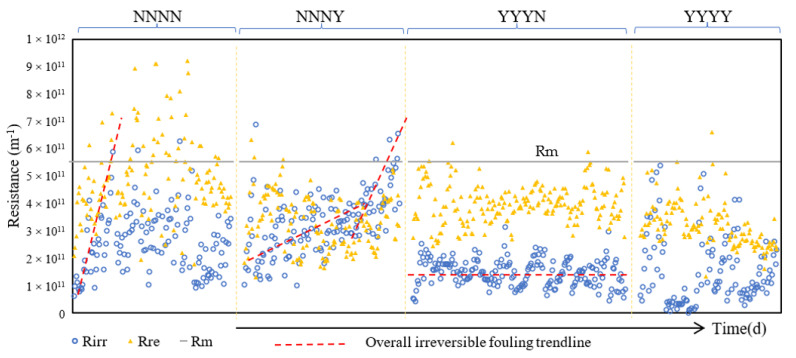
Fouling resistance variation versus time in different backwash conditions.

This section investigates the influence of chlorinated backwash on fouling composition. Figure 3 shows the variation of fouling resistance at the end of each filtration cycle in the four conditions. Membrane resistance (R_m_) was considered related to the initial permeability in each condition (650 L·m^−2^·h^−1^·bar^−1^) thus was constant at 5.51 × 10^11^ m^−1^ in all conditions. From Figure 3, the R_irr_ and R_re_ in condition NNNN increased rapidly between neighboring CEBs which can easily increase over R_m_. This situation can refer to Akhondi et al. [53] who confirmed the increase of fouling rate with periodic physical backwash under constant flux and dead-end filtration mode, and the fouling rate could be highly increased when the fouling achieves to a certain amount, together with poor reversibility and cleaning efficiency. On the contrary, the variation of R_irr_ and R_re_ in conditions with NaClO in backwashes (NNNY, YYYN, YYYY) were mostly under the R_m_. In actuality, it is important to limit the fouling deposition on the membrane, especially the R_irr_ on membrane, due to its negative impacts on hydraulic filtration performance and cleaning efficiency. In condition NNNY, the addition of NaClO in AB largely decreased R_irr_ and R_re_ compared to NNNN. Ignoring the filtration from the beginning to the first CEB, the overall R_irr_ variation can be separated into two parts, the slower increasing rate of overall R_irr_ from the 2nd to 5th day and the faster increasing rate of overall R_irr_ from the 5th to 7th day shown in Figure 3. In filtration from 2nd to 5th day, the overall R_irr_ increasing rate was in slow and steady growth during constant flux dead-end filtration. From each backwash sequence (four filtration cycles with CB, CB, CB, AB), the R_irr_ accumulated on the membrane was increased in the first three filtration cycles with CBs and was significantly decreased after an AB with NaClO which controlled the overall R_irr_ at a lower increasing rate. CB without NaClO could carry away partial cake foulant from the membrane surface but could not remove the smallest compounds adsorbed onto the membrane material effectively; when filtration restarts the residual foulant will adhere to the membrane surface again and accumulate continuously [45]. AB itself has powerful strength with air injection to loosen the foulant layer and to break and/or carry away the smaller particles blocked and absorbed into the membrane [41]. Then, after air injection, backwash with NaClO can inactivate the microorganisms and oxidize organic matters deposited on membrane. The cooperation of air flow and NaClO greatly improved the backwash efficiency on foulant cake removal. When the fouling resistance achieved to a certain value, the AB and CB cleaning efficiency was decreased and the accumulation of integral foulant on the membrane became faster than before. Thus, in filtration from 5th to 7th day, a higher overall R_irr_ rate was observed, and the fouling cake seemed hardly to be removed by CBs and unable to be controlled by ABs. 

The distribution of R_irr_ and R_re_ in condition YYYN were in the most stable variation, and R_irr_ was lower than R_re_ and lower than R_m_ (R_irr_ < R_re_ < R_m_). The addition of NaClO in CB showed an advantage on R_irr_ control from the beginning of the filtration compared to the previous conditions NNNN and NNNY. The variation of R_irr_ fluctuated slightly around the overall irreversible fouling trendline shown in Figure 3 with periodic ups and downs in YYYN which may be due to the periodic peak and trough periods of influent flow, secondary effluent quality, and temperature during a day, as stated in another paper [39]. The fouling rate was measured to be very low, and the fouling layer deposited in a filtration cycle seemed to be fully removed by backwashes. There is no more accumulation of R_irr_ after CBs in a backwash sequence in contrast to condition NNNY, thus the fouling removal efficiency of CB was largely improved by NaClO addition. Normally, the PES/PVP blended membranes shows superior hydrophilicity, anti-fouling, oxidative and thermal stability as well as good mechanical property [64,65]. However, when looking at condition YYYY, although the value of R_irr_ and R_re_ were both under R_m_, the variation of R_irr_ was in high volatility compared to YYYN. The R_irr_ sometimes increased very fast, and sometimes it was even lower than that in YYYN. Otherwise, the total fouling resistances (R_irr_ + R_re_) in conditions NNNY, YYYN, and YYYY were in similar ranges, around 4–7 × 10^11^ m^−1^ except the points around CEB in condition NNNY and YYYN. As the CEB is triggered at TMP ≥ 0.3 bar for 60 s, the corresponding total fouling resistance is at 1.41 × 10^12^ m^−1^ at 20 °C. The total fouling resistance in condition NNNN was more important, which resulted in frequent occurrence of CEB.

### 3.3. Reversibility of Physical Backwashes

**Figure 4:**
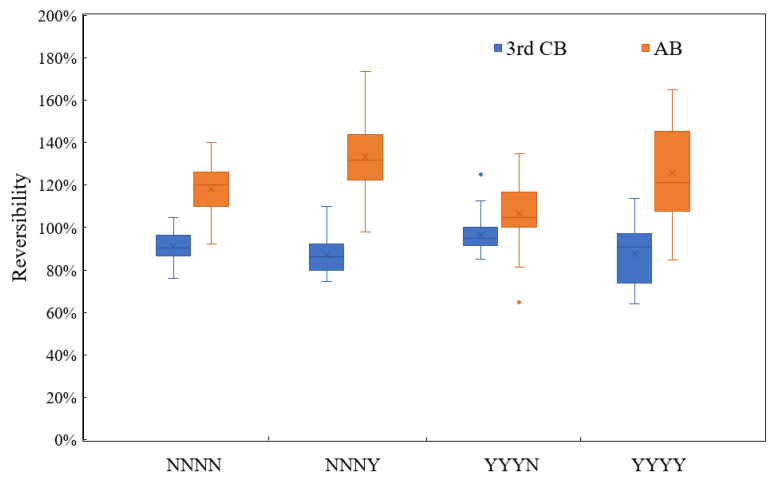
Reversibility ranges of air backwash (AB) and 3rd chemical backwash (CB) in different conditions.

From the previous analysis, the addition of NaClO in CB and AB improves the backwash cleaning efficiency, but it is not clear to know how much it can be improved. Therefore, it is necessary to compare the reversibility, which represents the backwash cleaning efficiency, in different backwash conditions. According to the membrane filtration model by Kalboussi et al. [66], the mass of the cake layer tends towards a constant value as the number of filtration cycles increase. According to the TMP variation in dead-end filtration with periodic backwashes [53,67], the fouling resistance at the end of each filtration cycle would be in increasing trend with filtration cycles continuing, therefore the fouling condition before AB is much closer to that before the third CB. Therefore, the fouling distribution before AB is much closer to the fouling distribution before the third CB compared to that before the first CB and second CB. Table 7 gives the reversibility of AB and the third CB ranges in different conditions, and Figure 4 shows the comparison between them. It can be confirmed that AB has a dominant position in reversibility compared to CB with or without NaClO, and even the addition of 10 mg Cl_2_ L^−1^ NaClO in CB could not reach the reversibility of AB without NaClO. Moreover, condition NNNY offered the highest average reversibility of AB (133 ± 16%) and the stable reversibility range (98–174%), and the second highest was condition YYYY. YYYN offered the lowest AB reversibility. The 10 mg Cl_2_·L^−1^ NaClO addition in AB improved the reversibility of AB by 15–18% on average through comparison in parallel conditions NNNN and NNNY, and in conditions YYYN and YYYY. However, the addition of 10 mg Cl_2_·L^−1^ NaClO in CB improved the reversibility of the third CB by 5% on average through comparison in parallel conditions NNNN and YYYN, and the third CB reversibility in condition NNNY and YYYY was very close to each other. The lowest reversibility of AB in condition YYYN may be because of its highest reversibility of the third CB compared to the other conditions. The cleaning performance of CB and AB mutually restricts and influences each other, as the reversibility in percentage is related to the previous filtration cycle. From the fouling resistance distribution in Section 3.2, the R_irr_ was controlled in the lowest ranges in YYYN compared to the others with chlorinated CB and indicated that the membrane was rather clean, therefore, the total foulant cake before AB in YYYN would be less than that in conditions NNNN and NNNY, which dealt with both the cake layer formed in one filtration cycle and accumulated foulant after the previous three CBs. Therefore, AB in YYYN did not exert its best removal ability because of the limited total fouling resistance, thus resulted in lower reversibility. On the other hand, although backwash condition YYYN largely improved the fouling management, there was still residual R_irr_ left on the membrane after chlorinated CB. However, non-chlorinated AB failed to remove these residual foulant which reflected its limitation on particular R_irr_ removal, while chlorinated AB in condition YYYY could achieve much lower value of R_irr_ if the sensitivity of the membrane to fouling is ignored.

**Figure 5:**
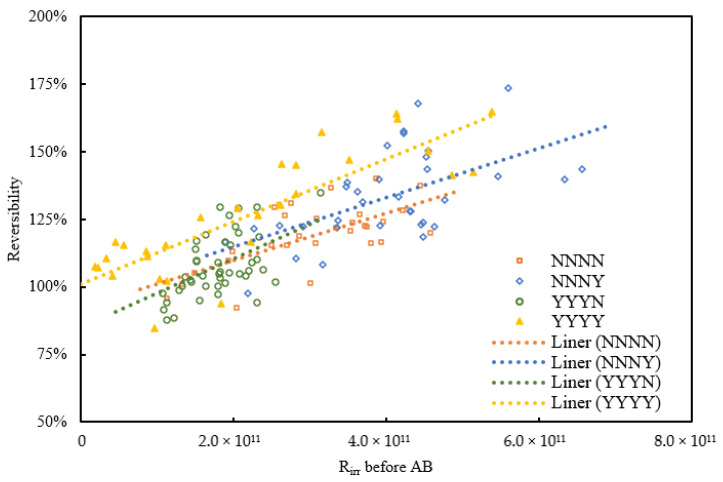
Reversibility of AB versus R_irr_ on the membrane just before AB.

**Figure 6:**
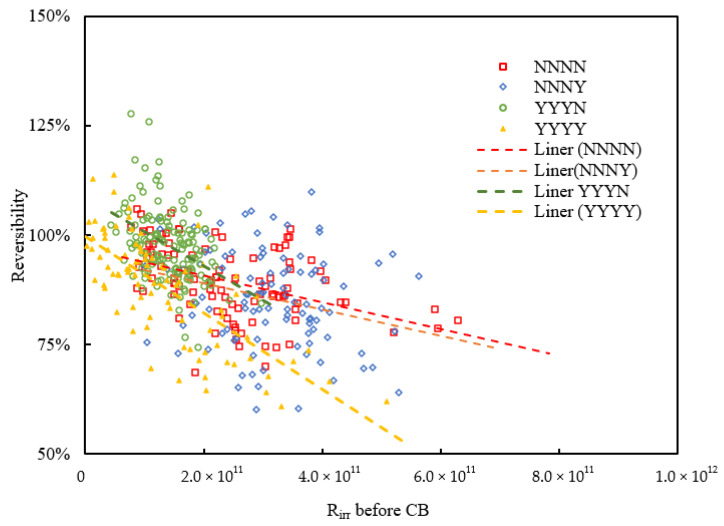
Reversibility of CB versus R_irr_ on the membrane just before CB.

To further investigate the impacts of irreversible fouling on cleaning efficiency by physical backwash, the relationship of backwash reversibility versus the R_irr_ on the membrane just before the corresponding AB or CB and the dynamic trendlines are shown in Figure 5 and Figure 6. The cases of CB in Figure 6 include the first CB, second CB, and third CB of all backwash sequences. The statistical test showed positive linear regression between AB reversibility and R_irr_ before AB in all conditions (*p*-value < 0.05) and showed significant negative linear regression on CB reversibility and R_irr_ before CB in condition NNNN, YYYN, and YYYY, while condition NNNY (*p* = 0.066) was not very significant. However, the data here were mainly shown to know the variation tendency (increase or decrease, not necessarily linearly) of the AB or CB reversibility versus total irreversible fouling resistance before the backwash. Therefore, the significances of both AB and CB under 90% confidential interval can be totally supported.

The reversibility of AB whether with or without NaClO was shown in positive correlation to R_irr_ from Figure 5, while the reversibility of CB with or without NaClO was shown in negative correlation to R_irr_ from Figure 6. This means that the cleaning capacity of AB will be increased with the increase of R_irr_ leading to better filtration performance, but the removal capacity of CB will be decreased with the increase of R_irr_ causing harsh filtration performance. From Figure 5, the increasing rates of AB reversibility with R_irr_ in all conditions were similar, the reversibility of AB under the same R_irr_ was in YYYY higher than NNNY, and higher than NNNN and YYYN. The addition of NaClO in AB indeed improved its cleaning efficiency as NNNY and YYYY were both in higher reversibility compared to NNNN and NNNY whatever the value of R_irr_.

From Figure 6, the reversibility of CB was decreased with the deposited R_irr_ increase and validated the higher performance of AB in comparison on CB. The slight decline of CB reversibility in conditions NNNN and NNNY was very close to each other and both conditions used non-chlorinated CB for cleaning. The CB of YYYN and YYYY showed higher reversibility with lower R_irr_ deposition, but the CB reversibility decreased faster with R_irr_ increase compared to condition NNNN and NNNY. On the one hand, the chlorinated CB resulted in higher reversibility that could better control R_irr_ in lower levels. On the other hand, although chlorinated CB was good at R_irr_ controlling, it had poor adaptability and lower reversibility when R_irr_ increased. Looking back to NNNN and NNNY, the cleaning efficiency of non-chlorinated CB was more stable and sustainable with R_irr_ variation. Condition YYYN had the best fouling resistance management, non-chlorinated AB resulted in lower reversibility which was much closer to the performance of chlorinated CB. Both conditions YYYN and YYYY showed faster decrease of CB reversibility with R_irr_ increase. From this point, condition NNNY is more recommended as the optimized backwash condition which showed relatively higher effective and stable reversibility variation both of AB and CB compared to other conditions.

### 3.4. Cake Layer Removal Efficiency by Backwash through Mass Balance

**Figure 7:**
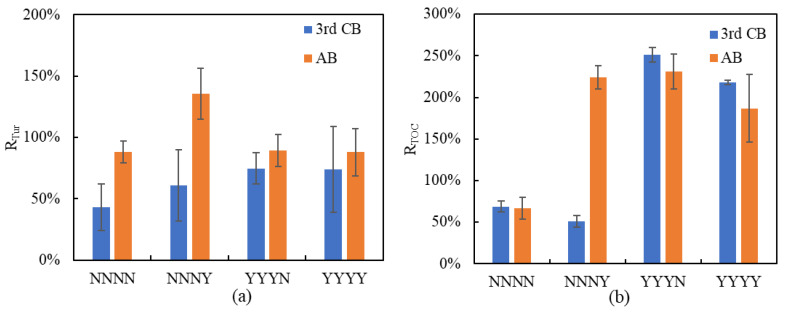
(**a**) Turbidity recovery rates of AB and 3rd CB, (**b**) TOC recovery rates of AB and 3rd CB.

In general, as the cake layer mass grows in proportion to the concentration of foulants in the feed, and to the total volume of feed passing through the membrane, the cake layer resistance is proportional to the mass accumulated on the membrane surface [67]. Table 8 shows the water quality of AB, third CB, UF feed, and UF permeate in different conditions, and Figure 7a,b is the turbidity and TOC removal rates by AB and third CB through mass balance in a filtration cycle.

Figure 7a shows that the percentage of turbidity recovered by the third CB is always below 80% which indicated there would be an increased foulant deposition of this residual turbidity after CB together with the new turbidity accumulation for the next filtration cycle. The turbidity removal capacity of the third CB was improved by 15–30% with 10 mg Cl_2_·L^−1^ NaClO addition compared to the non-chlorinated third CB. Additionally, the turbidity removal performance of AB was much better than CB, as the lowest removal rate was around 88% on average. The chlorinated AB in condition NNNY showed the highest, with 136% removal of turbidity from concentrate, which means the chlorinated AB completely removed the total turbidity accumulated in its own filtration cycle and removed part of the residual turbidity from the previous filtration cycles. The turbidity recovery performances of AB in conditions NNNN, YYYN, and YYYY were very similar, and the chlorinated AB in condition YYYY showed no improvement on turbidity removal. The removal efficiency of AB in condition NNNY was the highest compared to the other three conditions almost in similar efficiency.

From Figure 7b, it is obvious to see the high improvement of TOC recovery in conditions with NaClO compared to NNNN. In condition NNNN, the third CB and AB, both without NaClO, showed similar and low removal efficiency on TOC. TOC recovery rate by chlorinated AB was highly improved to more than 200% while by the third CB, it was only 50% in condition NNNY. Otherwise, the addition of NaClO in CB also greatly improved the TOC recovery rates as in conditions YYYN and YYYY. Through comparison, the addition of NaClO in CB showed higher removal capacity on TOC compared to turbidity removal.

Therefore, for TOC and turbidity recovery, when no NaClO is added in CBs (NNNN and NNNY), the AB, either with or without NaClO, will greatly improve the removal efficiency on turbidity thus compensates to the low removal efficiency of CB without NaClO. When adding NaClO in CBs (YYYN and YYYY), CB with NaClO will improve the removal efficiency on turbidity previous to AB, thus AB either with or without NaClO is proportionally less effective compared to CB with NaClO.

Based on the analysis on filtration permeability, fouling resistance distribution, backwash reversibility, and cake layer removal ability, it can be concluded that the addition of 10 mg Cl_2_·L^−1^ NaClO in AB and CB can greatly improve the overall filtration performance and cleaning efficiency. Through comparison, the condition NNNN was not suggested for operation because of the frequent CEB occurrence, fastest R_irr_ increase and poor cleaning efficiency by CB and AB. Condition YYYY was not recommended because of its potential damage on membrane material with most frequent contact with NaClO, unstable permeability and R_irr_ variation, and unstable cleaning efficiency of CB. YYYN was offered in the best fouling resistance control and permeability recovery, but the chlorinated CB showed lower reversibility when faced with higher R_irr_ on the membrane. Additionally, the addition of NaClO in condition YYYN will be higher that NNNY under the same operation period, which would increase the operating costs and may cause faster damage on membrane structures. Although the control of R_irr_ in condition NNNY was not as good as in condition YYYN, it was in slight and sustainable increase resulted in only 1 CEB occurrence in 6 days. Condition NNNY can be considered as the most cost-effective backwash condition in this study mainly due to less consumption of NaClO, sustainable and adaptable filtration performance, and less potential damage on the physicochemical characteristics of membrane. Therefore, the condition NNNY is recommended for long-term operation.

### 3.5. The Influence of NaClO Concentration on Air Backwash Efficiency of Condition NNNY

**Figure 8:**
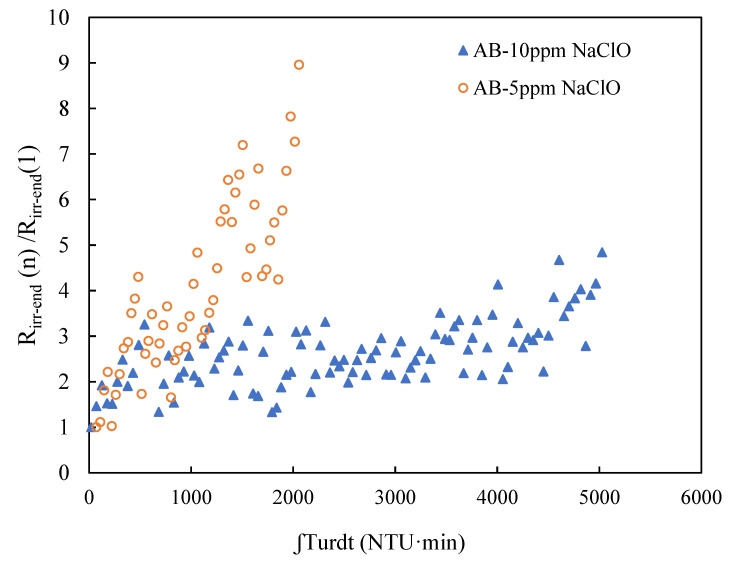
Variation of R_irr-end_ of filtration cycle (n) to the initial cycle (1) a function of the integral of turbidity vs. time.

As backwash sequence has been optimized in condition NNNY, it is necessary to further investigate the performance of NNNY condition with different concentrations of NaClO addition. The concentration of NaClO at 5 mg Cl_2_·L^−1^ and 10 mg Cl_2_·L^−1^ in condition NNNY are compared on the filtration performance. The long-term filtration results of 5 mg Cl_2_·L^−1^ NaClO condition has already been shown in another paper [39], of which the frequency of CEB occurrence was once in three days. In this study, the CEB occurred once in six days in NNNY with 10 mg Cl_2_·L^−1^ NaClO. To be compared, the variation of R_irr_ at the end of each filtration cycle in 5 mg Cl_2_·L^−1^ NaClO and 10 mg Cl_2_·L^−1^ NaClO conditions is shown in Figure 8. The Y axis R_irr-end_ (n)/R_irr-end_ (1) represents the increasing ration of R_irr-end_ during filtration, and X axis represents the foulant cake mass accumulation during filtration. Within 1000 NTU·min integration, the variation of R_irr-end_ (n)/R_irr-end_ (1) in condition NNNY with 5 mg Cl_2_·L^−1^ and 10 mg Cl_2_·L^−1^ of NaClO was closer to each other, and the ration of R_irr-end_ (n)/R_irr-end_ (1) was all under 5. After this, the R_irr_ increasing rate in 5 mg Cl_2_·L^−1^ NaClO condition was increased much faster than before 1000 NTU·min integration, while the variation in 10 mg Cl_2_·L^−1^ NaClO condition was continuously in sustainable and slight increase. The R_irr_ variation in condition NNNY with 5 mg Cl_2_·L^−1^ and 10 mg Cl_2_·L^−1^ NaClO was from 1.02 × 10^11^ m^−1^ to 9.17 × 10^11^ m^−1^, and from 1.35 × 10^11^ m^−1^ to 6.57 × 10^11^ m^−1^, respectively. The faster increase of R_irr-end_ after 1000 NTU·min integration in 5 mg Cl_2_·L^−1^ NaClO condition may be due to more complex structure of foulant cake layer and increased local flux due to loss of effective filtration area, as stated in Section 3.2 of condition NNNY. Higher concentration of NaClO at 10 mg Cl_2_·L^−1^ increased the chance to inactivate organisms, detach cake foulant, extend the time to form more complex fouling, and improve the load capacity on feed water quality. Therefore, the increase of NaClO concentration in condition NNNY indeed improved the AB cleaning efficiency and the overall R_irr_ control during filtration. It was found that 10 mg Cl_2_·L^−1^ NaClO is more effective on fouling control in condition NNNY.

## 4. Discussion

In this study, 10 mg Cl_2_·L^−1^ NaClO addition in backwash water can positively improve the filtration performance of a UF pilot plant. From Table 7 and Figure 4, the addition of NaClO had significant improvement on AB cleaning efficiency when comparing condition NNNN and NNNY, or comparing condition YYYN and YYYY, resulting in a 15–18% increase of reversibility. However, NaClO addition itself almost had less improvement on the third CB cleaning, with an average of 1–5% increasing of reversibility when comparing condition NNNN and YYYN, or condition NNNY and YYYY. In this case, it seems that air injection contributes more to enhance the NaClO-assisted backwash cleaning. In NaClO-assisted AB, air injection with strong shear force can break and dried the foulant layer on membrane, thus increasing contact area of foulant and chlorinated backwash water. The cooperation of air injection and NaClO oxidation on fouling highly improved the cleaning efficiency of backwash. Additionally, comparing the performance of AB and third CB in all conditions, the reversibility of AB (with or without NaClO) was completely higher than that of CB (with or without NaClO). Therefore, it seems that the effects of air injection on CB were more significant than NaClO assistance in CB water; this can be considered as AB without NaClO in this study has higher cleaning efficiency than CB with NaClO, see as in condition YYYN. Based on the comparison on efficiency of chemical cleaning and physical cleaning from Park et al. [68], extending contact time of chemical cleaning was one of the important factors on improvement of membrane permeability recovery compared to physical cleaning. In our study, the contact time of chlorinated backwash water through the membrane is 52 s in total. The disinfection and oxidation reactions between NaClO and cake foulants cannot be completely reacted in shorter contact time, thus the cleaning efficiency of chlorinated CB was limited in this study. In contrast, the physical assistance by air injection could greatly improve the cleaning efficiency. As was the case for Guigui et al. [69], air injection even at very low air velocity (0.08 m·s^−1^) could result in a high backwash efficiency, and normally, the duration of air injection does not need too long; less than 2 min is enough. Based on the above analysis, air injection assistance on CB (AB) indeed provides higher contributions on fouling removal than 10 mg Cl_2_·L^−1^ NaClO. However, the comparison conditions of AB and NaClO-assisted CB in this study could be completed with consideration of all impact factors. The impact factors include air injection duration, air velocity, NaClO concentration in backwash water, temperature, and NaClO contact time with membrane. Additionally, the co-effects among these factors are complex and could be investigated in the future. 

## 5. Conclusions

In conclusion, 10 mg Cl_2_·L^−1^ NaClO addition in backwash water is confirmed to greatly improve the overall filtration performance and backwash cleaning efficiency. During this experiment, the chronological order of conditions is NNNN, NNNY, YYYN, and finally YYYY. The type of backwash among all conditions is three CBs followed with one AB. N represents a backwash either CB or AB without NaClO and Y represents a backwash either CB or AB with NaClO. First, condition NNNY stands out from the other due to better control of R_irr_, less NaClO consumption in 10 years prediction, sustainable and adaptable filtration performance, and less potential damage on the physicochemical properties of the membrane. The second best condition is YYYN, which resulted in the best fouling control and permeability recovery, but it has higher potential to damage membrane structures under frequent NaClO addition. In all conditions, AB showed positive correlation of reversibility and R_irr_ increase, but CB showed negative correlation of reversibility and R_irr_ increase. Furthermore, even CB added with 10 mg Cl_2_·L^−1^ NaClO hardly exceeds the reversibility of AB without chlorine. These results reflect that AB itself, whether with or without NaClO, has high cleaning efficiency on fouling removal, and is especially better on R_irr_ control. Through comparison of 5 mg Cl_2_·L^−1^ and 10 mg Cl_2_·L^−1^ NaClO in condition NNNY, higher NaClO concentration increased the chance to inactivate organisms, detach cake foulant, extend the time to more complex fouling formation, and improve the load capacity on feed water quality. Therefore, condition NNNY with 10 mg Cl_2_·L^−1^ NaClO is mostly recommended for long-term operation in this study on secondary effluent treatment.

## Figures and Tables

**Table 1:** Studies on the combined physical backwash with low concentrated oxidants/disinfectants in backwash water.

No.	Backwash Types	Concentration	Backwash Time × Flux or TMP	Filtration Time × Flux	Feed Water	Membrane Process (Pore Size/Area)	Remarks	Ref.
1	NaClO-assisted backwash	23.8 mg Cl_2_·L^−1^	24 s/(207–241.5 kPa)	(15–30 min) × (34–102 L·m^−2^·h^−1^)	Tertiary treated wastewater	UF (150 kDa/1.9 m^2^)	● Frequent backwash with chlorine addition significantly improved membrane productivity, primarily due to enhanced foulant removal by organic oxidation and bio-growth control.	[34]
2	NaClO-assisted backwash	0.191 mg Cl_2_·L^−1^ (optimized)	15 min × 8.33 L L·m^−2^·h^−1^	12 h × 6 L·m^−2^·h^−1^	Synthetic municipal wastewater	MBR (0.01 μm/0.1 m^2^)	● NaClO backflush enhanced the detachment of biopolymers from the fouled membranes and enhanced the denitrification of MBR. ● Low level NaClO-assisted backflush has slight or few adverse effects on sludge and membranes.	[8]
3	NaClO-assisted backwash	0.953 mg Cl_2_·L^−1^ (optimized)	30 s × 30 L·m^−2^·h^−1^	9 min × 10 L·m^−2^·h^−1^	Domestic wastewater	anaerobic ceramic MBR (0.08 μm/0.08 m^2^)	● The biodegradability of organics in the wastewater and the microbial activities of biomass were improved with low level of NaClO-assisted backwash. ● High level of NaClO-assisted backwash deteriorated cell metabolism and led to excessive production of cell lytic products.	[9]
4	Backwash with chlorinated water	3 mg Cl_2_·L^−1^	2 min × 60 L·m^−2^·h^−1^	58 min × 20 L·m^−2^·h^−1^	Lake water/the Yangtze River water/micro-polluted water/municipal secondary effluent	UF (100 kDa/29.0 cm^2^)	● The use of chlorinated-water backwashing decreased the number of microorganisms in the biofouling layer, but increased the level of EPS; thus, the membrane fouling resistance decreased by 8.6%.	[10]
5	NaClO-assisted backwash	95.3 mg Cl_2_·L^−1^	1 h soaking	(TMP up to 40 kPa) × 20 L·m^−2^·h^−1^	Algal-rich water	UF (0.01 μm/0.025 m^2^)	● Among the tested cleaning reagents (NaOH, HCl, EDTA, and NaClO), 95.3 mg Cl_2_·L^−1^ NaClO exhibited the best performance (88.4% ± 1.1%) in removing the irreversible fouling resistance. ● The surface morphology of the fouled membrane almost recovered the original state of new membrane after cleaning with NaClO.	[35]
6	NaClO-assisted backwash (CEB)	284 mg Cl_2_·L^−1^ (optimized)	5.22 L·m^−2^·h^−1^	-	Synthetic mediumcontained (NH_4_)_2_SO_4_, NaNO_2_ and some trace elements	MBR (0.01 μm/0.05 m^2^)	● The best cleaning effect was evident at the NaClO concentration of 284 mg Cl_2_·L^−1^.	[38]

The units of chlorine used in different references were all uniformed to mg Cl_2_·L^−1^ in this paper, based on the relationship between NaClO and Cl_2_: 1 mg·L^−1^ NaOCl = 0.953 mg Cl_2_·L^−1^.

**Table 2:** UF membrane introduction.

Characteristics	Data
Material	Blended PES (polyethersulfone)
Pore size–MWCO	0.02 µm–200 kDa
Length	1.2 m
Internal diameter ID	0.9 mm
Number of channels	7
Filtration surface	9 m^2^
Volume of fibers	2.0 L
Maximum TMP	2.5 bar
pH tolerant value	1–13

**Table 3:** Feed water quality.

Parameters	Values
*E. coli* (CFU 100 mL^−1^)	(2.3 ± 1.9) × 10^4^
Enterococci (CFU 100 mL^−1^)	(9.3 ± 5.5) × 10^3^
Anaerobic sulphito-reducers (spores) (CFU 100 mL^−1^)	(9.6 ± 5.1) × 10^2^
Specific F-RNA bacteriophages (PFP 100 mL^−1^)	<30
COD (mgO_2_·L^−1^)	45 ± 21
TSS (mg·L^−1^)	12.1 ± 8
TOC (mgC·L^−1^)	6.6 ± 0.4
Turbidity (NTU)	1.9 ± 0.6
NH_4_^+^ (mgN·L^−1^)	3.8 ± 2.4
pH	7.5 ± 0.1

**Table 4:** Physical backwash parameters.

Steps	AB with Cl (RL A1)	AB without Cl (RL A2)	CB with Cl (RL C1)	CB without Cl (RL C2)
Step 1	Air injection into the fibers (2 min)	Air injection into the fibers (2 min)	-	-
Step 2	Decompression before standard backwash	Decompression before standard backwash	Decompression before standard backwash	Decompression before standard backwash
Step 3	Backwash top head (31 s)	Backwash top head (31 s)	Backwash top head (31 s)	Backwash top head (31 s)
Step 4	Backwash 2 heads (3 s)	Backwash 2 heads (3 s)	Backwash 2 heads (3 s)	Backwash 2 heads (3 s)
Step 5	Backwash bottom head (18 s)	Backwash bottom head (18 s)	Backwash bottom head (18 s)	Backwash bottom head (18 s)
Step 6	Backwash prefilter (12 s)	Backwash prefilter (12 s)	Backwash prefilter (12 s)	Backwash prefilter (12 s)
Permeate tank (volume)	T1 (29.25 L) + T2 (6.75 L)	T2 (36 L)	T1 (29.25 L) + T2 (6.75 L)	T2 (36 L)
Duration	184 s	184 s	64 s	64 s

**Table 5:** Backwash conditions of this study.

No.	CB	CB	CB	AB	Name
1	No Cl	No Cl	No Cl	No Cl	NNNN
2	No Cl	No Cl	No Cl	10 mg Cl_2_·L^−1^ Cl	NNNY
3	10 mg Cl_2_·L^−1^ Cl	10 mg Cl_2_·L^−1^ Cl	10 mg Cl_2_·L^−1^ Cl	No Cl	YYYN
4	10 mg Cl_2_·L^−1^ Cl	10 mg Cl_2_·L^−1^ Cl	10 mg Cl_2_·L^−1^ Cl	10 mg Cl_2_·L^−1^ Cl	YYYY

**Table 6:** The estimation of NaClO consumption and CT parameters of each condition in 10 years.

Name	Estimated Total CT in 10 Years (mg Cl_2_·L^−1^·h)	CT of Backwash in 10 Years (mg Cl_2_·L^−1^·h)	Equivalent Consumption of NaClO [gCl_2_·m^−3^ (Permeate)]	CEB Frequency	Maximum NaClO CT Value of UF Module
NNNN	60,833	0	272	Once per day	23,853 mg Cl_2_·L^−1^·day
NNNY	15,225	3058	175	Once in 5 days	
YYYN	19,630	9490	414	Once in 6 days	
YYYY	24,820	10,544	546	Once in 5 days	

**Table 7:** Reversibility ranges of backwash in different conditions.

Condition	3rd CB			AB		
	Min	Max	Mean ± SD	Min	Max	Mean ± SD
NNNN	76%	102%	91 ± 7%	92%	140%	118 ± 12%
NNNY	75%	104%	86 ± 8%	98%	174%	133 ± 16%
YYYN	85%	125%	96 ± 7%	81%	135%	107 ± 12%
YYYY	64%	114%	87 ± 14%	85%	165%	125 ± 21%

**Table 8:** The turbidity and TOC in different types of backwash water and UF feed and permeate.

Condition	Water Types	Turbidity (NTU)	TOC (mg·L^−1^)
All conditions	UF feed	1.9 ± 0.9	6.6 ± 0.4
	UF permeate	0.4 ± 0.3	6.3 ± 0.3
NNNN	AB	20.2 ± 2	9.3 ± 1
	3rd CB	10.1 ± 2	9.4 ± 0.6
NNNY	AB	30.9 ± 6	16.4 ± 2
	3rd CB	14.1 ± 4	8.6 ± 0.6
YYYN	AB	20.5 ± 3	18.9 ± 3
	3rd CB	17.2 ± 2	17.6 ± 2
YYYY	AB	20.2 ± 9	14.7 ± 6
	3rd CB	17.0 ± 8	16.1 ± 0.4

## Data Availability

Not applicable.
